# Effectiveness of Electrical Stimulation Combined with Pelvic Floor Muscle Training on Female Sexual Dysfunction with Overactive Bladder: A Randomized Controlled Clinical Trial

**DOI:** 10.3390/jpm14090938

**Published:** 2024-09-03

**Authors:** JunJie Piao, Dongho Shin, MyeongKeon Moon, SaeWoong Kim, WoongJin Bae

**Affiliations:** 1 Department of Urology, College of Medicine, The Catholic University of Korea, Seoul 03083, Republic of Korea; pjj920911@gmail.com (J.P.); eds8813@naver.com (D.S.); ksw1227@catholic.ac.kr (S.K.); 2 Catholic Integrative Medicine Research Institute, The Catholic University of Korea, Seoul 06591, Republic of Korea; 3 Green Medicine Co., Ltd., Busan 48307, Republic of Korea; 4 China-Korea Joint Research Center for Male Reproductive and Sexual Medicine, Institute of Urology, Tianjin Medical University, Tianjin 301700, China

**Keywords:** electrical stimulation, overactive bladder, pelvic floor

## Abstract

(1) Background: Female sexual dysfunction (FSD) and overactive bladder (OAB) are prevalent disorders that significantly impair women’s quality of life. While pelvic floor muscle training (PFMT) is a well-established treatment for OAB, its effectiveness for FSD remains uncertain. Recent studies suggest that intravaginal electrical stimulation (IVES) may enhance the effects of PFMT. Therefore, this study aimed to evaluate the efficacy of combining IVES with PFMT to improve sexual function and bladder control in women with OAB and FSD. (2) Methods: A total of 40 women with FSD and OAB as determined by the Female Sexual Function Index (FSFI) and Overactive Bladder Symptom Score (OABSS) were randomized into two groups: group 1, the intravaginal electrical stimulation group (IVESG) (*n* = 20), and group 2, the placebo control group (CG) (*n* = 20). Patients in both groups received PFMT during the trial, and the IVESG underwent 10 weeks of electrical stimulation. The primary outcome was the change in the FSFI score from baseline to week 4 and week 10. The secondary outcome was a comparison of the OABSS values at week 4 and week 10 of the trial. (3) Results: After treatment, the IVESG scores for the total FSFI, sexual arousal and orgasm, increased significantly (*p* < 0.05). After 10 weeks of treatment, the OABSS values for IVESG were significantly lower (*p* < 0.01). After 10 weeks of treatment, the percentage of patients with mild OAB in the IVESG and CG increased by 53.33% and 60%, respectively, while the percentage of patients with both moderate and severe OAB decreased, especially in the IVESG, where patients with severe OAB were non-existent after 10 weeks of treatment. (4) Conclusions: IVES combined with PFMT improved objective and subjective measures of FSD and OAB. There was a significant improvement in FSD (including arousal and orgasmic domain scores). This study concludes that IVES is an effective conservative treatment.

## 1. Introduction

Sexual dysfunction is a common problem in the general population [1]. Sexual behavior affects women’s emotional and mental health. Therefore, the importance of clinical treatment for FSD is increasing [2]. The psychological fear of leaking urine during sexual activity is thought to contribute to sexual dysfunction [3]. Therefore, FSD is partly due to urine leaking [4]. Sexual dysfunction was more common in women than in men [5,6], accounting for 30 to 50% of women, and the incidence increases with age [7]. A previous study found that 57% of middle-aged women complained of sexual dysfunction and urinary frequency [8]. In women, diabetes, heart diseases, urologic diseases, and chronic diseases were important risk factors for sexual dysfunction [5].

A retrospective study reported that the treatment of FSD is complex because it includes multiple complex symptoms, which respond differently to different treatment modalities [9]. Various therapies, including behavioral therapies, have been used to treat FSD and have shown reasonable efficacy in enhancing libido [10,11,12,13,14]. Pelvic floor rehabilitation therapy, including PFMT, vaginal exercises, and anal biofeedback [15], has shown good efficacy in enhancing libido and sensitivity, improving vaginal looseness, and urinary incontinence.

PFMT such as strengthening, endurance, coordination, stretching, and relaxation of the pelvic floor muscles is expected to improve sexual dysfunction and urge incontinence [16]. IVES is a device with a probe with electrodes specially inserted into the vagina, generating a controlled electric current and regulating the intensity, frequency, and duration of the electrical impulses according to the patient’s specific situation and tolerance. In recent years, the efficacy of electrical stimulation and biofeedback physical therapy for female sexual dysfunction has been reported. Intravaginal biofeedback (IVBF) and IVES have been used in clinical practice. Emma Bendana, et al. evaluated IVBF/IVES in women with pelvic floor muscle (PFM) spasms associated with urinary symptoms. Fifty-two women received IVBF/IVES with a mean symptom improvement rate of 64.5% [14,15,16]. In Korea, 32 patients with stress urinary incontinence were also treated with eight sessions of transcutaneous electrical stimulation once a day, 5 to 6 times a week, to evaluate the Female Sexual Function Index scores and PFM parameters, but no vaginal penetration electrical stimulation device was used [16].

We investigated the effectiveness of a low-frequency therapeutic device to improve FSD and OAB. Although this medical device has not been used to treat FSD, it has been used in patients with urinary incontinence [17]. Considering the potential relevance of improved PFM function for both FSD and OAB, we conducted this trial.

## 2. Materials and Methods

### 2.1. Participants

This randomized controlled trial was conducted from December 2020 to December 2021 at a urology clinic in Seoul, South Korea. Women with frequent visits for female sexual dysfunction (FSD) and overactive bladder (OAB) were contacted to inquire about their participation in this study. Women who expressed interest in participating were provided with verbal and written information and were required to give informed consent before group assignment and baseline assessment. The study adhered to the principles outlined in the CONSORT 2010 guidelines to ensure transparency and rigor in the design, conduct, and reporting of the trial.

We employed double-blind parallel randomization with a comparable number of subjects as in similar studies [16,17] and pre-calculated the required sample size using G*Power 3.1.3 (University of Trier, Trier, Germany) with a power of 0.80, an alpha level of 0.05, and an effect size of f = 0.917. This calculation suggested that more than 6 subjects were needed in each group, and considering a 20% dropout rate, at least 15 subjects per group. Based on the inclusion and exclusion criteria, the final number of volunteers was determined to be 40. Volunteers were randomly assigned into two groups according to a simple randomization procedure (1:1 ratio by computer): the IVESG (*n* = 20) and the CG (*n* = 20).

The inclusion criteria included women aged 20 years or older, assessed using structured questions based on the FSFI and the OABSS. Women who had previously received physiotherapy for FSD and those with stress urinary incontinence were excluded. Inclusion criteria also included a total FSFI score of less than 26 [16] and the presence of lower urinary tract symptoms such as urinary urgency, frequency, nocturia, and urge incontinence. Exclusion criteria included latex allergies, vaginal or urinary infections, pelvic organ prolapse greater than grade II [18], inability to perform voluntary PFMT, cognitive or neurological disorders, uncontrolled hypertension, inability to carry out the evaluation or treatment, ongoing hormonal therapy, and the use of a pacemaker or metal rod implantation [19,20,21].

### 2.2. Outcome Measures

All evaluations were meticulously conducted by a single-blinded and experienced physiotherapist. Initially, all participants underwent a comprehensive physical examination followed by a detailed interview concerning their medical history. Both the IVESG and CGs were assessed before and after treatment, focusing on the primary outcome measure of FSFI score and the secondary outcome measure of OABSS.

The FSFI is a comprehensive questionnaire designed to evaluate sexual function in women. This instrument comprises 19 items, encompassing six key domains of sexual function: sexual desire, arousal, lubrication, orgasm, satisfaction, and pain during sexual intercourse. Each item is rated on a scale ranging from 0 to 5, with higher scores reflecting superior sexual function. The cumulative FSFI score, which aggregates the six domain scores, ranges from 2 to 36 [22].

The OABSS has been established as a prevalent tool for assessing Overactive Bladder (OAB) syndrome. Based on OABSS results, we categorized patients into three levels of OAB severity: mild (OABSS 1–5), moderate (OABSS 6–11), and severe (OABSS ≥ 12), comparing these classifications pre- and post-treatment [23].

### 2.3. Treatment Protocol

Prior to device distribution, patients received comprehensive instructions on their proper use. Patient guides, detailing the modes (intensity, duration, and current amplitude) tailored for each patient, were distributed. The MK-000A Dr. Lady device (Buheung Medical, Seoul, Republic of Korea) was utilized in this study. All participants were instructed in skills and strategies, provided with an informational pamphlet, and engaged in daily pelvic floor muscle training (PFMT). The PFMT regimen included approximately 30 near-maximal contractions, each lasting 5–10 s, followed by a 10 s pause, adjusted according to the woman’s pelvic floor muscle function [24].

Following protocols established in previous studies [25], in the IVES group, the electrical frequency was set to 40 Hz for 12 min. The action time was divided into specified cycles: 3 s resting cycles with increasing intensity, 4 s intensity maintenance, 3 s decreasing intensity, and 5 s rest periods. The maximum amplitude reached 22 V (±30%) and the maximum current was 6 mA (±30%). Concurrently, heat therapy at 35 °C to 40 °C and micro-vibration were administered. Before the electrical stimulation, the probe vibrated to indicate the start signal. In the control group, only a vibration signal was present at the beginning, with no further stimulation. Patients underwent treatment twice daily, with each session lasting 12 min, for a duration of 10 weeks. During probe insertion, a small amount of lubricant gel was applied to the probe surface. Both sets of instrument programs were pre-set to run automatically upon pressing the start button on the device.

### 2.4. Statistical Analysis

Sexual intercourse was allowed and encouraged during treatment. Student’s *t*-test or Mann–Whitney U-test was used for comparison of continuous variables between two groups. The chi-square or Fisher’s exact test was used to compare qualitative variables. In each group, change from baseline was analyzed using a paired *t*-test in the parametric data or Wilcoxon signed rank test in the nonparametric data. Statistical analyses were performed by SAS version 9.4 (SAS Institute Inc., Cary, NC, USA), and *p* values < 0.05 were considered statistically significant.

## 3. Results

### 3.1. Demographic Characteristics

**Figure 1:**
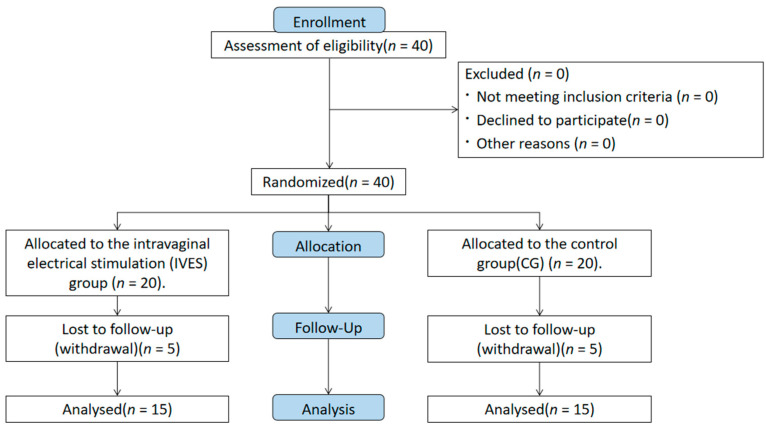
Participants flow diagram.

Among the forty women, five (12.5%) from the control group were excluded due to the absence of a final assessment caused by health issues. Similarly, five participants (12.5%) in the IVES group were excluded due to reported vaginal infections. Thirty volunteers completed the study and were included in the analysis. The demographics and medical and gyneco-obstetric backgrounds of the patients are presented in Figure 1 and Table 1, showing no significant differences between the groups at baseline.

### 3.2. Primary Outcomes

**Figure 2:**
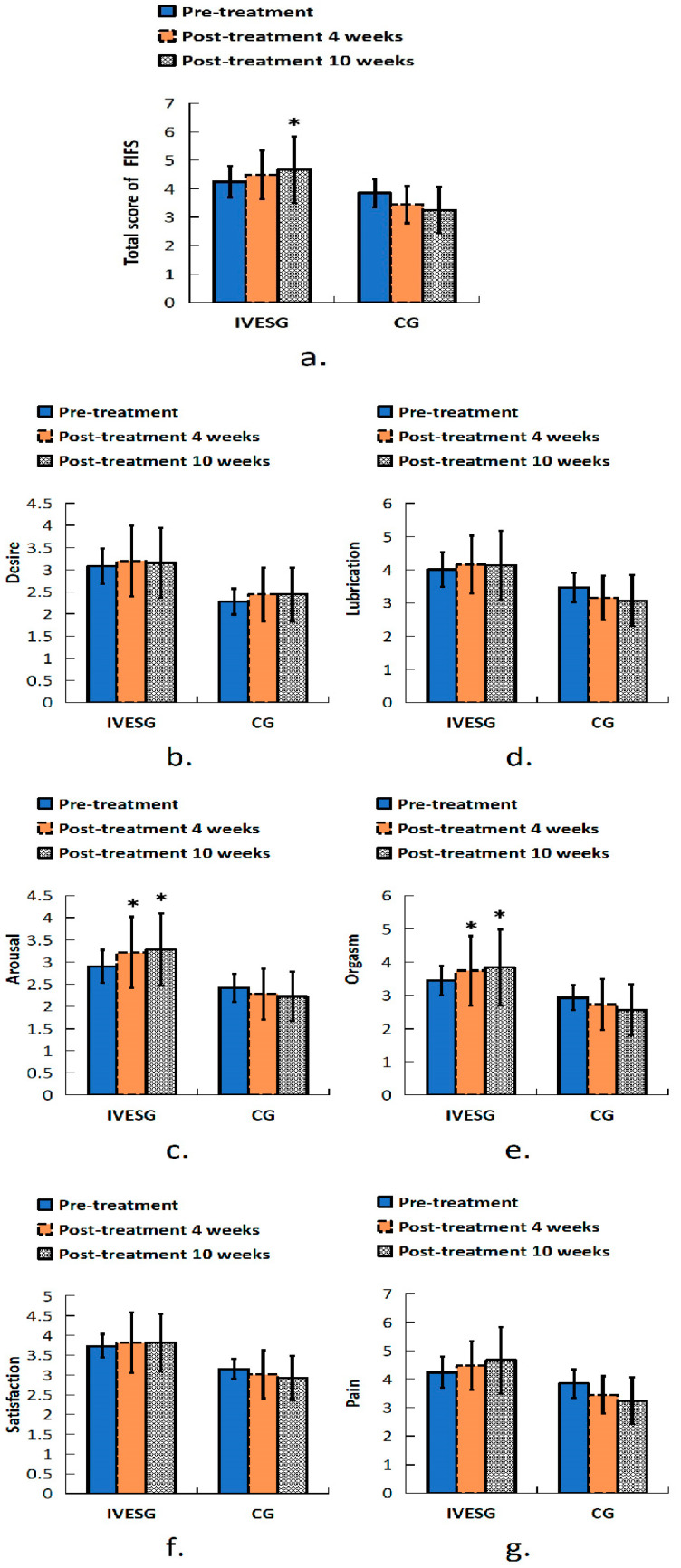
Total FSFI score of women with FSD and OAB. (**a**) Sexual function in all domains before and after 4 and 10 weeks of treatment: (**b**) desire, (**c**) arousal, (**d**) lubrication, (**e**) orgasm, (**f**) satisfaction, (**g**) pain. * Indicates significant differences between pre-treatment and post-treatment.

In the FSFI assessment, after 10 weeks of treatment, the total FSFI score in the IVESG showed a significant increase compared to baseline (*p* = 0.0394), whereas no significant difference was observed in the CG. In the arousal domain, a statistically significant increase was observed in the IVESG after 4 and 10 weeks of treatment (*p* = 0.0234 and *p* = 0.0117, respectively). The orgasm domain also showed improvement in the IVESG, with a significant increase in scores after 4 and 10 weeks of treatment (*p* = 0.0313). No significant changes were observed in the CG (Table 2, Figure 2). 

### 3.3. Secondary Outcomes

**Figure 3:**
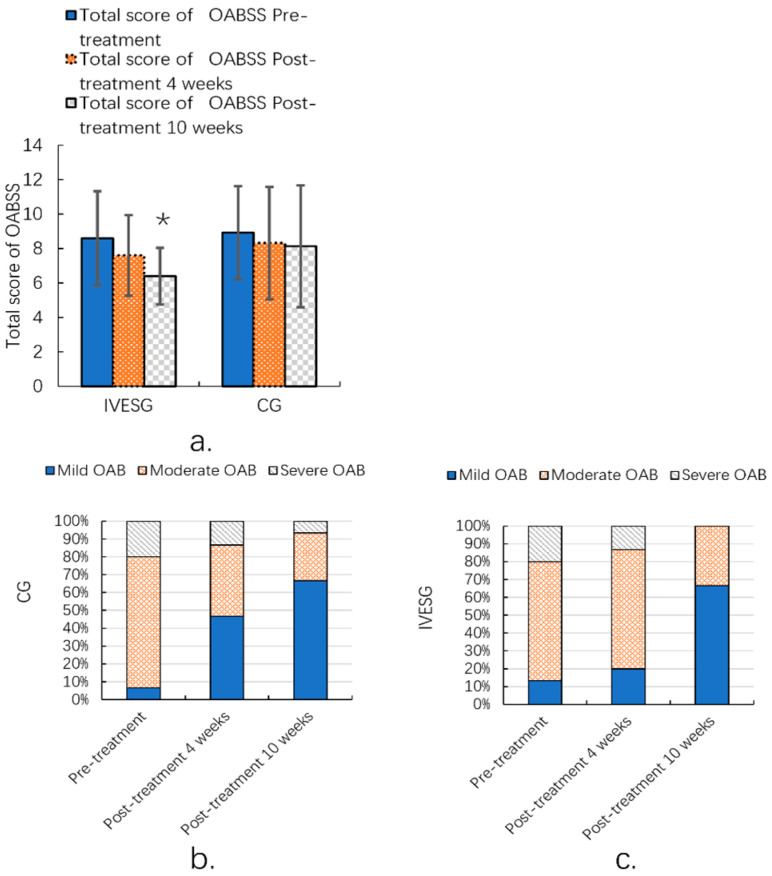
Total OABSS of women with FSD and OAB (**a**); OABSS grade ratio before and after 10 weeks of IVESG treatment (**b**,**c**). * Significant differences between pre-treatment and post-treatment.

After 10 weeks of treatment, the total OABSS in the IVESG showed a statistically significant improvement compared to pre-treatment levels (*p* = 0.0038). In the IVESG, the proportion of patients with mild OAB increased significantly from 13.33% before treatment to 66.67% after 10 weeks. The proportion of patients with moderate OAB decreased from 66.67% to 33.33%, and those with severe OAB decreased dramatically from 20.00% to 0.00%. In the CG, the proportion of patients with mild OAB increased from 6.67% to 66.67%. The proportion of patients with moderate OAB decreased from 73.33% to 26.67%, and the proportion of patients with severe OAB decreased from 20.00% to 6.67%. Both groups demonstrated signs of improvement, with an increase in the proportion of patients with mild OAB and a decrease in the proportion of those with moderate and severe OAB. This was particularly notable in the IVESG, where the severe cases disappeared completely. (Table 3, Figure 3).

## 4. Discussion

In this study, we sought to compare the effects of IVES combined with PFMT on FSD and OAB in women. At the end of the study, patients in the IVESG showed improvements in the total FSFI score and domain scores, especially in arousal and orgasm. There was also a significant improvement in OAB symptoms. IVES improved sexual arousal and orgasm. Overall, our findings are consistent with previous studies of IVES for FSD and OAB.

Sexual dysfunction is a complex problem involving different aspects of the body, mind, and social culture. Pelvic muscle spasm in dysfunction is a defensive reflex against threatening situations. Therefore, in addition to psychotherapy, the rehabilitation of pelvic muscles plays an important role in the treatment of dysfunction. IVES strengthens the pelvic floor muscles [20,21]. In our previous study, the effects of electrical stimulation and vibration on skeletal muscle increased simultaneously [17]. This electrical stimulation and micro-vibration could increase the systolic force acting on muscle fibers. In addition, simultaneous heat therapy increased blood flow and reduced muscle pain caused by pelvic congestion. The simultaneous transmission of three stimuli or types of energy can maximize treatment effect by inducing synergy [25]. Studies have shown that low-frequency electrical stimulation could improve pain, the pain threshold, and relieve local pain [21]. This explained why IVES reduced pain in patients with FSD and dyspareunia. In addition, electrical stimulation induced a gradual desensitization to pain [21]. Therefore, patients benefited from long-term improvements in pain control.

In a study of 31 women with lower urinary tract symptoms, almost all parameters of the King’s Health Questionnaire (KHQ) showed improvement in symptoms after 8 weeks of treatment. The low-frequency electrical stimulation device was effective in improving urinary incontinence, and the improvement in OAB symptoms was more pronounced with longer treatment duration [25].

In a previous study, 42 FSD women were randomly allocated to IVES and placebo groups. Pelvic floor muscles evaluations and the FSFI questionnaire were conducted after baseline and coursework. The probe was inserted and a medium-frequency (50 Hz) alternating current was applied with an operating cycle of 5 s and a rest of 5 s. The main end-to-end indicator was improvements in the FSFI scores. The pelvic floor muscle evaluation was based on the PERFECT scheme. Both the IVESG and CG showed significant improvements in the total FSFI scores. The results showed that the total FSFI scores were improved in the IVESG, including arousal, desire, climax, and satisfaction. Similarly, the areas that improved in the CG were desire, arousal, and climax [26]. Our research showed that IVES was effective in terms of arousal and orgasm. This treatment has been found to be effective in improving sexual quality of life.

Our study presents several limitations that need to be acknowledged, each of which could impact the validity and generalizability of the results.

Brief Duration of Follow-Up: One of the most significant limitations of our study is the relatively short duration of the follow-up. The limited follow-up period may have insufficiently captured the long-term outcomes and potential delayed effects of the intervention or condition being studied. The short-term follow-up can obscure the sustainability of effects and the potential emergence of long-term side effects or benefits. As a result, our findings may not fully reflect the enduring impact of the intervention, potentially leading to an overestimation or underestimation of its effectiveness. Unfortunately, extending the follow-up period was not feasible due to constraints such as time, resources, and participant availability.

Influence of Pelvic Floor Muscle Training (PFMT): Another potential weakness of the study is the influence of pelvic floor muscle training (PFMT) on the results. PFMT was incorporated as a component of the intervention, and its effects could confound the results related to the primary research question. This confounding factor makes it difficult to isolate and evaluate the specific impact of the primary intervention alone. The potential overlap in benefits between PFMT and the primary intervention could skew the results, making it challenging to determine the precise contribution of each component. We did not address this limitation more thoroughly due to the complexity of separating the effects and the practical difficulties involved in controlling for PFMT’s influence within the study design.

Limited Sample Size: The relatively small number of participants enrolled in the study is another notable limitation. A limited sample size can reduce the statistical power of the study, increasing the risk of Type II errors (i.e., failing to detect a true effect when one exists) and decreasing the ability to generalize the findings to a broader population. Small sample sizes can also lead to increased variability and less reliable results. Our study’s sample size constraints were due to factors such as recruitment challenges and budgetary limitations. Despite these challenges, we proceeded with the study as planned to gather preliminary data, with the understanding that future research would need to address this limitation with larger and more diverse samples.

In summary, while these limitations have influenced our study’s outcomes and interpretations, they highlight important areas for future research. Addressing these limitations in subsequent studies will be crucial for validating our findings and advancing the understanding of the topic.

Despite these limitations, this study yielded promising results, as significant improvements were observed using standardized and validated criteria. Both the primary and secondary outcome indicators exhibited notable improvement. These findings are encouraging and suggest the potential effectiveness of the intervention. To our knowledge, there are existing studies on intravaginal electrical stimulation (IVES) in the scientific literature that report its application in the overactive bladder (OAB) population; however, there remains a notable deficiency in research focused on female sexual dysfunction (FSD) treatment. Future research should prioritize refining the study design, expanding the sample size, and establishing a higher level of evidence by comparing IVES with sham treatments and the current therapies for both FSD and OAB.

## 5. Conclusions

IVES with PFMT significantly improved objective and subjective measures of FSD and OAB. Noteworthy improvements were observed in the area of female sexual dysfunction, particularly in arousal and orgasm. Thus, IVES is an effective and conservative treatment for FSD and OAB.

## Figures and Tables

**Table 1:** Demographic and clinics characteristics of the study participants (*n* = 30).

	IVESG (*n* = 15)	CG (*n* = 15)	*p*-Value ^a^
Age (years)	52.60 ± 8.53	57.33 ± 8.62	0.1418
Height (cm)	159.83 ± 4.09	157.65 ± 5.52	0.2307
Weight (kg)	58.33 ± 9.69	57.37 ± 9.05	0.7797
BMI (kg/m^2^)	28.28 ± 4.345	28.26 ± 4.16	0.454
Number of deliveries	2.74 ± 1.36	2.45 ± 1.41	0.35
Vaginal deliveries	1.02 ± 1.14	1.26 ± 1.36	0.432
Duration of symptoms (y)	6.7 ± 3.24	7.3 ± 4.46	0.398
Menopause	10 (66.67%)	6 (40.00%)	0.1432

Data presented as mean ± standard deviation; IVESG, intravaginal electrical stimulation group; CG, control group. ^a^ Student’s *t*-test.

**Table 2:** Values of the (FSFI) Female Sexual Function Index domains for the groups.

Outcome	Comparison	Group	Mean ± SD	*p*-Value ^a^	Comparison	Group	Mean Difference ± SD	*p*-Value ^b^
FSFI total score	Baseline	IVESG	21.39 ± 3.99	0.1198				
		CG	18.08 ± 4.97					
	Post-treatment 4 weeks	IVESG	22.61 ± 4.62	0.0176 #	Baseline vs. Post-treatment 4 weeks	IVESG	1.22 ± 6.10	0.0515
		CG	17.05 ± 7.17			CG	−1.03 ± 8.72	0.848
	Post-treatment 10 weeks	IVESG	22.90 ± 4.40	0.0186 #	Baseline vs. Post-treatment 10 weeks	IVESG	1.51 ± 5.94	0.0394 *
		CG	16.47 ± 8.70			CG	−1.61 ± 10.02	0.4639
Desire	Baseline	IVESG	3.08 ± 0.84	0.0257 #				
		CG	2.28 ± 1.02					
	Post-treatment 4 weeks	IVESG	3.20 ± 0.98	0.0391 #	Baseline vs. Post-treatment 4 weeks	IVESG	0.12 ± 1.29	0.50
		CG	2.44 ± 1.03			CG	0.16 ± 1.45	0.50
	Post-treatment 10 weeks	IVESG	3.16 ± 0.86	0.0364 #	Baseline vs. Post-treatment 10 weeks	IVESG	0.08 ± 1.20	0.75
		CG	2.44 ± 1.03			CG	0.10 ± 1.20	0.50
Arousal	Baseline	IVESG	2.90 ± 0.77	0.1617				
		CG	2.42 ± 1.04					
	Post-treatment 4 weeks	IVESG	3.22 ± 0.93	0.0376 #	Baseline vs. Post-treatment 4 weeks	IVESG	0.32 ± 1.21	0.0234 *
		CG	2.28 ± 1.38			CG	−0.14 ± 1.73	0.9844
	Post-treatment 10 weeks	IVESG	3.28 ± 0.88	0.0260 #	Baseline vs. Post-treatment 10 weeks	IVESG	0.38 ± 1.17	0.0117 *
		CG	2.22 ± 1.51			CG	−0.20 ± 1.83	0.4511
Lubrication	Baseline	IVESG	4.00 ± 0.70	0.0493 #				
		CG	3.46 ± 0.74					
	Post-treatment 4 weeks	IVESG	4.16 ± 0.82	0.0359 #	Baseline vs. Post-treatment 4 weeks	IVESG	0.16 ± 1.08	0.2813
		CG	3.16 ± 1.52			CG	−0.30 ± 1.69	1.0000
	Post-treatment 10 weeks	IVESG	4.14 ± 0.83	0.1074	Baseline vs. Post-treatment 10 weeks	IVESG	0.14 ± 1.09	0.4922
		CG	3.07 ± 1.80			CG	−0.40 ± 1.95	0.4375
Orgasm	Baseline	IVESG	3.44 ± 0.77	0.1068				
		CG	2.93 ± 0.89					
	Post-treatment 4 weeks	IVESG	3.74 ± 0.69	0.0182 #	Baseline vs. Post-treatment 4 weeks	IVESG	0.30 ± 1.03	0.0313 *
		CG	2.72 ± 1.36			CG	−0.21 ± 1.63	1.0000
	Post-treatment 10 weeks	IVESG	3.84 ± 0.64	0.0111 #	Baseline vs. Post-treatment 10 weeks	IVESG	0.40 ± 1.00	0.0313 *
		CG	2.56 ± 1.63			CG	−0.37 ± 1.86	0.2813
Satisfaction	Baseline	IVESG	3.73 ± 0.58	0.0140 #				
		CG	3.15 ± 0.71					
	Post-treatment 4 weeks	IVESG	3.81 ± 0.69	0.0393 #	Baseline vs. Post-treatment 4 weeks	IVESG	0.08 ± 0.90	0.5501
		CG	3.01 ± 1.00			CG	−0.14 ± 1.23	0.7188
	Post-treatment 10 weeks	IVESG	3.81 ± 0.66	0.0342 #	Baseline vs. Post-treatment 10 weeks	IVESG	0.08 ± 0.88	0.5951
		CG	2.93 ± 1.35			CG	−0.22 ± 1.53	0.4453
Pain	Baseline	IVESG	4.24 ± 1.00	0.2638				
		CG	3.84 ± 0.98					
	Post-treatment 4 weeks	IVESG	4.48 ± 1.12	0.0634	Baseline vs. Post-treatment 4 weeks	IVESG	0.24 ± 1.50	0.2188
		CG	3.44 ± 1.76			CG	−0.40 ± 2.01	0.6250
	Post-treatment 10 weeks	IVESG	4.67 ± 1.19	0.0417 #	Baseline vs. Post-treatment 10 weeks	IVESG	0.43 ± 1.55	0.0859
		CG	3.25 ± 2.00			CG	−0.59 ± 2.23	0.2813

Data are presented as mean ± standard deviation (SD); IVESG: intravaginal electrical stimulation group; CG: control group; * Indicates significant differences between pre-treatment and post-treatment; # Indicates significant differences versus the control group. ^a^ Mann–Whitney U-test; ^b^ Wilcoxon test.

**Table 3:** The value of the total score of the Overactive Bladder Symptom Score (OABSS) domains for the groups.

Outcome	Comparison	Group	Mean ± SD	*p*-Value ^a^	Comparison	Group	Mean Difference ± SD	*p*-Value ^b^
OABSS	Baseline	IVESG	8.60 ± 2.72	0.7393				
		CG	8.93 ± 2.71					
	Post-treatment 4 weeks	IVESG	7.60 ± 2.35	0.7536	Baseline vs. Post-treatment 4 weeks	IVESG	−1.00 ± 3.59	0.1367
		CG	8.33 ± 3.27			CG	−0.60 ± 4.25	0.2866
	Post-treatment 10 weeks	IVESG	6.40 ± 1.64	0.1011	Baseline vs. Post-treatment 4 weeks	IVESG	−2.20 ± 3.18	0.0038 *
		CG	8.13 ± 3.54			CG	−0.80 ± 4.46	0.2170

Data presented as mean ± standard deviation; IVESG, intravaginal electrical stimulation group; CG, control group; * Significant differences between pre-treatment and post-treatment. ^a^ Mann–Whitney U-test. ^b^ Wilcoxon test.

## Data Availability

Our research data are all reflected in the original results.
